# Alzheimer’s Disease as a Disorder of Neuroimmune Dysregulation

**DOI:** 10.3390/neurolint18020037

**Published:** 2026-02-20

**Authors:** Gonzalo Emiliano Aranda-Abreu, Fausto Rojas-Durán, María Elena Hernández-Aguilar, Deissy Herrera-Covarrubias, Luis Roberto Tlapa-Monge, Sonia Lilia Mestizo-Gutiérrez

**Affiliations:** 1 Instituto de Investigaciones Cerebrales, Universidad Veracruzana, Xalapa 91190, Veracruz, Mexico; frojas@uv.mx (F.R.-D.); elenahernandez@uv.mx (M.E.H.-A.); dherrera@uv.mx (D.H.-C.); roberto.tlapa@outlook.com (L.R.T.-M.); 2 Facultad de Ciencias Químicas, Universidad Veracruzana, Xalapa 91190, Veracruz, Mexico; smestizo@uv.mx

**Keywords:** neuroinflammation, Alzheimer’s disease, microglia, astrocytes, synaptic loss, tau pathology

## Abstract

Alzheimer’s disease (AD) is traditionally defined by Amyloid-β (Aβ) plaques and tau neurofibrillary tangles, yet these proteinopathies alone fail to explain disease heterogeneity, progression, and cognitive decline. Emerging evidence identifies chronic neuroinflammation as a central integrator that converts molecular pathology into synaptic failure and neurodegeneration. In this context, Aβ acts as a danger-associated molecular pattern that activates microglial and astrocytic immune programs through receptors such as TREM2, TLRs, and RAGE, leading to inflammasome activation, cytokine release, and oxidative stress. These responses pathologically re-engage developmental complement pathways (C1q–C3–CR3), driving excessive synaptic pruning that correlates more closely with cognitive impairment than neuronal loss. Reactive astrocytes further amplify dysfunction by impairing glutamate and potassium homeostasis, promoting excitotoxic and metabolic stress, while inflammatory glia facilitate prion-like tau propagation via extracellular vesicles. Concurrent neurovascular inflammation disrupts blood–brain barrier integrity and cerebral perfusion, reinforcing immune-metabolic failure. Importantly, neuroinflammatory biomarkers (GFAP, sTREM2, YKL-40, cytokines, complement, and TSPO-PET) provide dynamic readouts of disease activity and therapeutic response. Together, these findings position AD as a disorder of failed immune resolution and support precision immunomodulatory and pro-resolving therapies aimed at restoring neuroimmune homeostasis rather than merely removing protein aggregates.

## 1. Introduction

Alzheimer’s disease (AD) has long been defined by the accumulation of extracellular amyloid-β (Aβ) plaques and intracellular neurofibrillary tangles composed of hyperphosphorylated tau [1]. For decades, these proteinopathies have been considered the primary drivers of neurodegeneration, forming the basis of dominant pathogenic models and therapeutic strategies [2]. However, growing evidence from genetics, neuroimaging, biomarker studies, and experimental models has revealed that neither amyloid nor tau alone sufficiently explains the spatial progression, clinical heterogeneity, or rate of cognitive decline observed in patients [3]. Instead, AD is increasingly recognized as a disorder of neuroimmune dysregulation in which chronic neuroinflammation acts as a key determinant of disease evolution [4].

Neuroinflammatory responses are an intrinsic component of normal brain physiology [5]. Microglia and astrocytes continuously monitor the neural environment, remove cellular debris, regulate synaptic remodeling, and coordinate tissue repair following injury [6]. In the early stages of AD, these glial responses are initially protective, promoting amyloid clearance, limiting oxidative damage, and supporting neuronal survival [7]. However, in the aging brain, repeated exposure to amyloid aggregates, tau pathology, metabolic stress, and vascular dysfunction progressively alters the set point of glial cells, shifting them toward maladaptive and self-sustaining inflammatory states [8]. Once this transition occurs, neuroinflammation ceases to function as a repair mechanism and instead becomes a powerful pathological amplifier.

This shift from protective to chronic neuroinflammation represents a fundamental turning point in Alzheimer’s disease [4]. Rather than simply reflecting the burden of plaques and tangles, inflammatory signaling actively converts protein aggregation into widespread synaptic failure, network disorganization, and neurodegeneration [9]. Activated microglia re-engage developmental programs of synaptic pruning through the complement system, leading to the inappropriate elimination of functional synapses—an event that correlates more closely with cognitive decline than amyloid load itself [10]. In parallel, reactive astrocytes lose their ability to regulate extracellular glutamate, potassium, and energy metabolism, creating conditions that favor excitotoxicity and network instability. Together, these glial alterations erode the integrity of neuronal circuits long before overt neuronal death becomes apparent [11].

Neuroinflammation also plays a central role in shaping the spatiotemporal propagation of tau pathology. Emerging evidence indicates that tau is not merely a passive intracellular aggregate but a biologically active signal that can be released, taken up, and transmitted between neurons in a prion-like manner [12]. Inflammatory microglia and astrocytes facilitate this process by secreting tau-containing extracellular vesicles, by incompletely degrading internalized tau, and by producing cytokines such as interleukin-1β and tumor necrosis factor-α that activate tau-phosphorylating kinases [13,14]. Through these mechanisms, neuroinflammation does not simply respond to tau pathology—it actively accelerates its spread across vulnerable brain networks.

Beyond the parenchyma, chronic neuroinflammation extends to the neurovascular unit, linking proteinopathy to vascular dysfunction. Inflammatory activation of endothelial cells and pericytes disrupts blood–brain barrier integrity, allowing plasma proteins, immune mediators, and peripheral immune cells to enter the brain [15]. These vascular changes further amplify local inflammatory responses and impair cerebral perfusion, creating a feed-forward loop in which immune dysregulation, metabolic stress, and protein aggregation reinforce one another. This convergence of neuroinflammation and vascular pathology helps explain why cerebrovascular dysfunction is a powerful predictor of disease progression and clinical severity in AD [16].

Critically, the persistence of neuroinflammation in Alzheimer’s disease is driven not only by pathological stimuli but also by aging-related changes in immune regulation. Microglial priming, astrocytic senescence, mitochondrial dysfunction, and impaired resolution pathways collectively lower the threshold for inflammatory activation while weakening the mechanisms that normally restore homeostasis [17]. As a result, even modest pathological insults can trigger exaggerated and prolonged inflammatory responses, locking the brain into a state of chronic immune activation that progressively undermines neural resilience [18].

Importantly, neuroinflammation in Alzheimer’s disease should not be viewed as a purely brain-confined process. Systemic inflammation and age-related immune remodeling (“inflammaging”) profoundly influence the central nervous system through humoral signaling, endothelial activation, and blood–brain barrier permeability [19]. Peripheral cytokines, metabolic stressors, and immune mediators can prime microglia and astrocytes, lowering the threshold for exaggerated inflammatory responses once cerebral pathology emerges [20]. In parallel, age-dependent impairment of the glymphatic system reduces the clearance of damage-associated molecular patterns (DAMPs), including but not limited to amyloid-β species, further sustaining immune activation [21]. Within this framework, central neuroinflammation arises from a dynamic interaction between peripheral immune states, vascular integrity, and impaired clearance mechanisms, rather than from isolated brain pathology alone.

**Figure 1:**
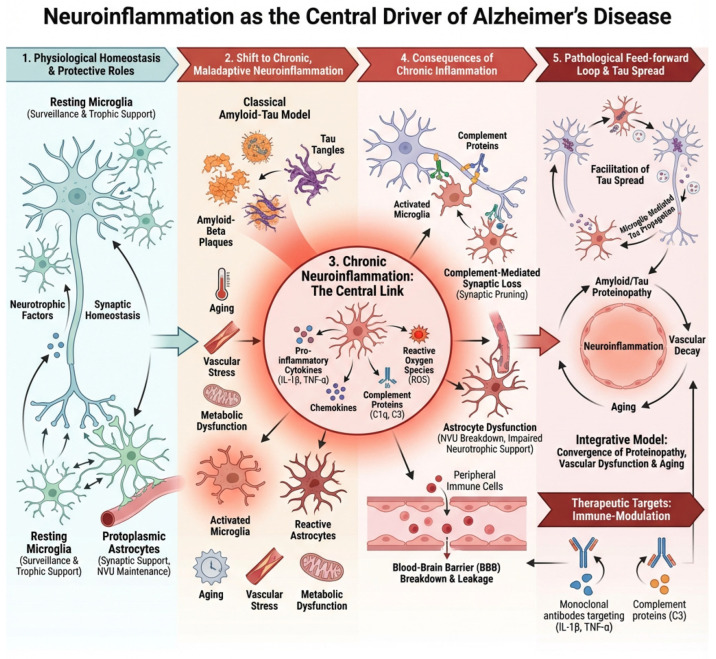
Neuroinflammatory axes driving Alzheimer’s disease pathology. This schematic summarizes five interconnected axes of neuroinflammation that contribute to the initiation, amplification, and progression of Alzheimer’s disease (AD): (1) Amyloid-β as an innate immune trigger, whereby soluble oligomeric Aβ acts as a danger signal that activates microglia and astrocytes; (2) Microglial activation and phenotypic reprogramming, encompassing the shift from homeostatic to disease-associated states that can be both protective and neurotoxic; (3) Astrocytic reactivity and metabolic dysfunction, including glial scar formation, impaired glutamate clearance, and altered neuronal support; (4) Complement-mediated synaptic pruning and synaptopathy, in which aberrant reactivation of developmental complement pathways leads to excessive synapse loss; and (5) Neurovascular and peripheral immune interactions, highlighting blood–brain barrier dysfunction, vascular inflammation, and infiltration of peripheral immune mediators that further sustain chronic neuroinflammation. Together, these axes form a self-reinforcing network that links immune dysregulation to synaptic failure and neurodegeneration in AD.

In this context, neuroinflammation should be viewed not as a secondary epiphenomenon of Alzheimer’s pathology but as a central integrator that transforms molecular lesions into systems-level failure. By amplifying synaptic loss, destabilizing neural networks, promoting tau propagation, and disrupting the neurovascular environment, chronic inflammation becomes a decisive force driving neurodegeneration [22]. Understanding Alzheimer’s disease through this neuroimmune lens provides a unifying context that connects genetic risk, protein aggregation, vascular dysfunction, and systemic aging, and it offers critical opportunities for the development of biomarkers and therapies aimed at restoring immune balance rather than simply removing protein aggregates, Figure 1.

Neuroinflammation in Alzheimer’s disease is increasingly understood as part of a broader systemic immune landscape [23]. Peripheral inflammatory conditions—including metabolic syndrome, obesity, insulin resistance, and cardiovascular disease—expose the brain to sustained cytokine signaling and promote microglial priming [24]. Altered lipid metabolism and chronic low-grade inflammation can bias microglia toward pro-inflammatory phenotypes and impair resolution pathways [25].

The gut–brain axis has emerged as another relevant contributor. Dysbiosis of the intestinal microbiota can enhance systemic inflammation, alter short-chain fatty acid signaling, and increase blood–brain barrier permeability, thereby influencing central immune activation. Endocrine changes, including glucocorticoid dysregulation and age-related decline in sex hormones, further modulate inflammatory tone and glial responsiveness [26].

Lifestyle factors such as sleep disruption, chronic stress, and physical inactivity may impair glymphatic clearance, exacerbate vascular dysfunction, and reinforce immune imbalance. These systemic contributors underscore that Alzheimer’s disease cannot be viewed solely as an isolated cerebral disorder but rather as the result of dynamic interactions between central vulnerability and peripheral immune and metabolic states [27].

Importantly, neuroinflammation in Alzheimer’s disease should not be conceptualized as a process confined to the brain, nor as one that necessarily originates within the central nervous system [23]. Increasing evidence indicates that systemic inflammation and age-related immune remodeling can precede and shape central neuroimmune responses. Peripheral inflammatory conditions, including metabolic syndrome, cardiovascular disease, chronic infections, and age-associated “inflammaging,” influence microglial priming through circulating cytokines, endothelial activation, and alterations in blood–brain barrier permeability [28]. In this context, neuroinflammation emerges as the central nervous system manifestation of a broader immunological imbalance rather than an isolated cerebral phenomenon. This systemic-to-central coupling helps explain interindividual variability in disease onset and progression and underscores the importance of considering Alzheimer’s disease within an integrated neuroimmune–vascular framework.

## 2. Amyloid Pathology as a Danger Signal Driving Neuroinflammation

Among the multiple factors that initiate and sustain neuroinflammation in Alzheimer’s disease (AD), amyloid-β (Aβ) occupies a unique position as both a pathological hallmark and a potent innate immune stimulus [29]. While amyloid plaques have traditionally been considered the primary toxic entities in AD, it is now widely recognized that soluble and oligomeric forms of Aβ are the principal immunologically active species [30]. These assemblies behave as damage-associated molecular patterns (DAMPs), alerting the brain’s innate immune system to cellular stress and protein misfolding [31]. Through this mechanism, Aβ does not simply accumulate passively but actively engages and reprograms glial cells, initiating the neuroinflammatory cascade that ultimately amplifies neurodegeneration.

Microglia and astrocytes express a broad repertoire of pattern-recognition receptors that enable them to detect Aβ as a signal of danger. Among these, Toll-like receptors (TLR2 and TLR4) [32], the scavenger receptor CD36 [33], the receptor for advanced glycation end products (RAGE) [34], and the lipid-sensing immune receptor TREM2 [35] are particularly important. Binding of oligomeric Aβ to these receptors triggers intracellular signaling pathways that converge on transcriptional and inflammasome-mediated inflammatory programs [36]. While such responses are initially aimed at clearing amyloid and restoring tissue homeostasis, persistent exposure to Aβ progressively shifts glial cells toward chronically activated, dysfunctional states.

One of the most critical pathways engaged by Aβ is the NLRP3 inflammasome, a multiprotein complex that serves as a molecular platform for the activation of interleukin-1β (IL-1β) and interleukin-18 [37]. Oligomeric Aβ promotes NLRP3 activation through multiple mechanisms, including lysosomal destabilization following phagocytosis, mitochondrial dysfunction, and the generation of reactive oxygen species [36]. Once activated, NLRP3 drives the maturation and release of IL-1β, a cytokine that profoundly alters neuronal and glial physiology. IL-1β enhances tau phosphorylation, disrupts synaptic plasticity, and reinforces inflammatory gene expression, thereby creating a feed-forward loop in which amyloid-induced inflammation perpetuates its own activation [38].

At the cellular level, Aβ-mediated signaling through TLRs, CD36, RAGE, and TREM2 pushes microglia away from their homeostatic surveillance phenotype toward reactive, disease-associated states [39]. These microglia exhibit increased phagocytic activity but also produce high levels of tumor necrosis factor-α (TNF-α), IL-1β, nitric oxide, and reactive oxygen and nitrogen species (ROS/RNS) [40]. Although these mediators are effective in killing pathogens and degrading debris, in the context of chronic exposure to Aβ they become neurotoxic. Oxidative and nitrosative stress damages synaptic proteins, impairs mitochondrial function, and destabilizes neuronal membranes, making neurons increasingly vulnerable to excitotoxic and metabolic insults [41].

Genetic background critically modulates how amyloid pathology is interpreted by the innate immune system. Among genetic risk factors, apolipoprotein E (APOE) isoforms exert a particularly strong influence on microglial metabolism, inflammatory bias, and disease trajectory [42]. Human studies, animal models, and cultured glial systems consistently show that APOE4 promotes a lipid-dysregulated, pro-inflammatory microglial phenotype characterized by impaired phagocytic efficiency and defective inflammatory resolution, whereas APOE2 is associated with more homeostatic and reparative immune responses [43]. These isoform-dependent effects indicate that amyloid toxicity is not intrinsic to the peptide itself but emerges from genetically biased immune interpretation. Thus, APOE genotype acts as a critical modifier that determines whether amyloid-triggered inflammation remains adaptive or becomes neurotoxic [44].

Astrocytes, which are activated both directly by Aβ and indirectly through microglial cytokines, further amplify this inflammatory environment. Reactive astrocytes lose their capacity to efficiently buffer glutamate and potassium, altering neuronal excitability and network stability [45]. They also secrete additional inflammatory mediators and acute-phase proteins, reinforcing glial–glial and glial–neuronal signaling loops that sustain inflammation. Thus, what begins as a localized immune response to amyloid aggregates becomes a system-wide disruption of synaptic and metabolic homeostasis [11].

Importantly, this inflammatory response is not simply proportional to amyloid burden. Instead, it reflects the brain’s interpretation of Aβ as a persistent danger signal. In genetically and age-primed brains—particularly in individuals carrying risk alleles such as APOE4 or TREM2 variants—the threshold for immune activation is lowered, and the resolution of inflammation is impaired [46].

Genetic risk factors further shape how amyloid pathology is interpreted by the innate immune system. Among these, apolipoprotein E (APOE) isoforms exert a profound influence on microglial metabolism, inflammatory bias, and disease trajectory [47,48]. Experimental and human studies consistently demonstrate that APOE4 promotes a lipid-dysregulated, pro-inflammatory microglial phenotype with impaired phagocytic efficiency and defective resolution, whereas APOE2 is associated with more homeostatic and reparative immune responses [49]. These isoform-dependent effects are observed across human post-mortem tissue, animal models, and cultured glial systems, underscoring that amyloid toxicity is not intrinsic to the peptide itself but emerges from genetically biased immune interpretation. Thus, genetic background acts as a critical modifier that determines whether amyloid-triggered inflammation remains adaptive or becomes neurotoxic [50,51].

As a result, even relatively modest amounts of oligomeric Aβ can elicit exaggerated and prolonged inflammatory responses that far exceed their direct neurotoxic effects [52].

**Figure 2:**
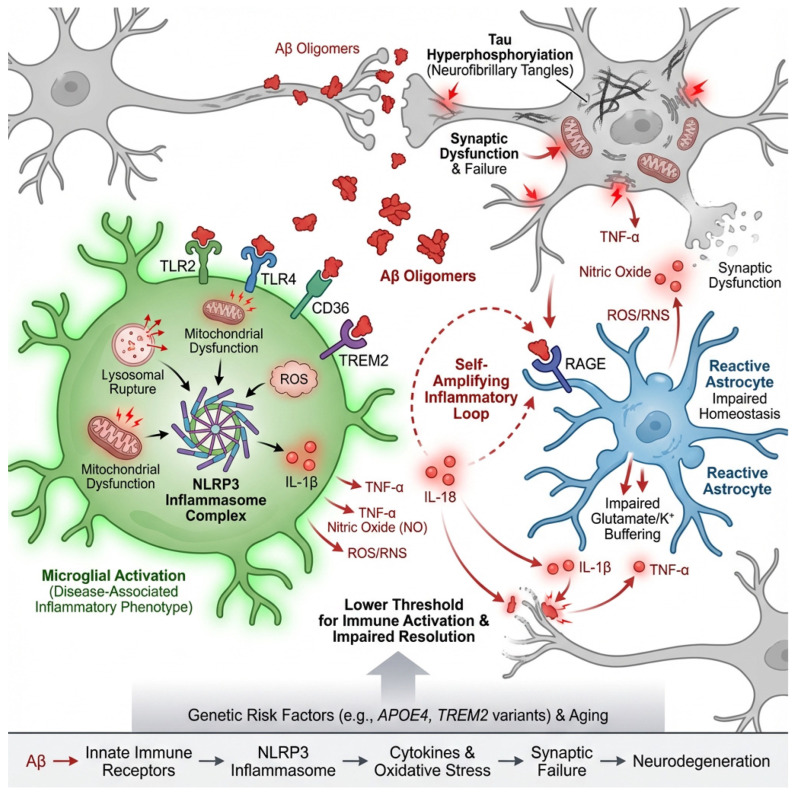
Amyloid-β as a danger signal driving neuroinflammation in Alzheimer’s disease. Oligomeric and fibrillar amyloid-β (Aβ) act as damage-associated molecular patterns (DAMPs) that are sensed by microglia and astrocytes through pattern-recognition receptors, including TLR2/4, CD36, RAGE, and TREM2. Engagement of these receptors triggers intracellular signaling cascades that activate inflammatory transcriptional programs and the NLRP3 inflammasome. Phagocytosis of Aβ induces lysosomal stress, mitochondrial dysfunction, and reactive oxygen species (ROS) production, leading to NLRP3 assembly and the maturation of IL-1β and IL-18. Released cytokines amplify glial reactivity, promote tau phosphorylation, disrupt synaptic plasticity, and create a feed-forward inflammatory loop. Microglia shift from a homeostatic to a disease-associated phenotype characterized by increased phagocytosis and release of TNF-α, IL-1β, and ROS/RNS, while reactive astrocytes impair glutamate and potassium buffering and secrete additional inflammatory mediators. Together, these processes convert localized amyloid pathology into widespread synaptic dysfunction, network instability, and progressive neurodegeneration.

Through these mechanisms, amyloid pathology acts not merely as a static deposit but as a dynamic immunological trigger that reshapes the cellular environment of the brain. By driving microglial and astrocytic reactivity, activating the NLRP3 inflammasome, and inducing the release of cytokines and oxidative mediators, Aβ converts localized protein misfolding into widespread synaptic dysfunction and network instability. This process represents one of the earliest and most powerful routes by which neuroinflammation is initiated and sustained in Alzheimer’s disease, setting the stage for tau propagation, vascular dysfunction, and progressive neurodegeneration [53,54], as shown in Figure 2.

## 3. Mechanisms Linking Neuroinflammation to Neurodegeneration

Neurodegenerative diseases such as Alzheimer’s disease (AD), Parkinson’s disease (PD), frontotemporal dementia (FTD), and amyotrophic lateral sclerosis (ALS) are no longer viewed as purely neuronal disorders. Instead, they are now recognized as disorders of dysfunctional cellular ecosystems in which chronic neuroinflammation plays a causal and self-amplifying role [55]. In this context, glial cells, the complement system, vascular elements, and immune signaling pathways interact to progressively erode synaptic integrity, neuronal metabolism, and network stability. Several convergent mechanistic axes explain how inflammation is converted into structural neurodegeneration [56].

### 3.1. Complement-Mediated Synaptopathy and Loss of Plasticity

One of the most powerful links between neuroinflammation and neurodegeneration is the aberrant reactivation of the complement system in the adult brain. During development, complement proteins such as C1q and C3 mark weak or unnecessary synapses for elimination by microglia, allowing circuit refinement [57,58]. In the aging and diseased brain, this developmental program is pathologically re-engaged.

Reactive astrocytes and microglia upregulate C1q in response to amyloid-β, misfolded tau, α-synuclein, and pro-inflammatory cytokines [59]. C1q binds to synaptic membranes, initiating the classical complement cascade and generating C3 fragments that opsonize synapses [60]. Microglia expressing complement receptor 3 (CR3) then phagocytose these tagged synapses. Importantly, this occurs before overt neuronal death and preferentially targets highly plastic glutamatergic synapses [61].

This complement-driven synaptic pruning produces an early and profound loss of functional connectivity. Long-term potentiation is impaired, dendritic spines retract, and neural networks become destabilized [62,63]. Cognitive decline in Alzheimer’s disease and network failure in other tauopathies correlate more strongly with synapse loss than with neuronal death itself, placing complement-mediated synaptopathy at the center of inflammation-driven neurodegeneration.

### 3.2. Astroglial Dysfunction, Excitotoxicity, and Ionic Collapse

Astrocytes are essential regulators of synaptic homeostasis. They clear extracellular glutamate through transporters such as EAAT2 (GLT-1 in rodents) [64], buffer extracellular potassium via Kir4.1 channels [65], and supply neurons with metabolic substrates. During chronic neuroinflammation, astrocytes transition into reactive phenotypes that profoundly disrupt these functions [66].

Pro-inflammatory cytokines such as TNF-α, IL-1β, and IFN-γ downregulate EAAT2 expression and impair glutamate uptake [67]. As a result, glutamate accumulates in the synaptic and extrasynaptic space, chronically activating NMDA and AMPA receptors. This leads to excessive calcium influx, mitochondrial overload, oxidative stress, and ultimately excitotoxic neuronal injury [68].

Simultaneously, reactive astrocytes lose their capacity to buffer extracellular K^+^, leading to neuronal depolarization and hyperexcitability. This ionic dysregulation further enhances glutamate release and synaptic firing, creating a vicious cycle of network instability. In diseases such as ALS and AD, loss of astrocytic EAAT2 is an early and consistent pathological feature, linking glial inflammation directly to neuronal death [69,70].

### 3.3. Reactive Glia as Facilitators of Tau Propagation

Neurodegenerative diseases characterized by tau pathology exhibit prion-like propagation of misfolded tau through neural circuits. Neuroinflammation strongly accelerates this process. Microglia and astrocytes, rather than merely responding to tau pathology, actively participate in its spread [71,72,73].

Activated microglia internalize tau aggregates and release them in exosomes or extracellular vesicles, allowing pathological tau to move between neurons [74]. Inflammatory signals enhance microglial phagocytosis and vesicle release, increasing tau mobility across brain regions. Astrocytes also internalize tau and can release it in a seeding-competent form, further amplifying network-level pathology [75].

In addition, cytokines and complement activation alter synaptic and axonal membranes, increasing their permeability and vulnerability to tau entry. Thus, neuroinflammation not only fails to contain tau pathology but actively converts it into a system-wide degenerative process [76].

### 3.4. Neurovascular Unit Dysfunction and Inflammatory Hypoperfusion

Neurodegeneration unfolds within the context of a disrupted neurovascular unit, composed of endothelial cells, pericytes, astrocytic endfeet, and neurons. Chronic inflammation damages each of these components.

Endothelial cells exposed to TNF-α, IL-6, and reactive oxygen species lose tight junction integrity, leading to blood–brain barrier (BBB) breakdown. Plasma proteins such as fibrinogen and albumin leak into the brain, activating microglia and astrocytes and further amplifying inflammation. Pericyte degeneration and astrocytic endfoot dysfunction impair capillary regulation, producing chronic hypoperfusion [77,78].

Microhemorrhages, capillary stalls, and reduced oxygen delivery lead to neuronal energy failure, impaired protein clearance, and enhanced tau and amyloid aggregation. Thus, neuroinflammation converts the brain’s vascular system from a support structure into a driver of metabolic and inflammatory stress [79,80].

### 3.5. Failure of Resolution and Persistent NF-κB Signaling

In healthy tissues, inflammation is self-limited and actively resolved by specialized pro-resolving lipid mediators (SPMs) such as resolvins, protectins, and maresins. In neurodegenerative disease, this resolution phase fails [81].

Aging, APOE4, and chronic microglial activation reduce the production and signaling of pro-resolving mediators, allowing inflammatory pathways such as NF-κB to remain chronically active. NF-κB drives the sustained expression of cytokines, complement proteins, iNOS, and inflammasome components, locking glial cells into a toxic phenotype [43].

**Figure 3:**
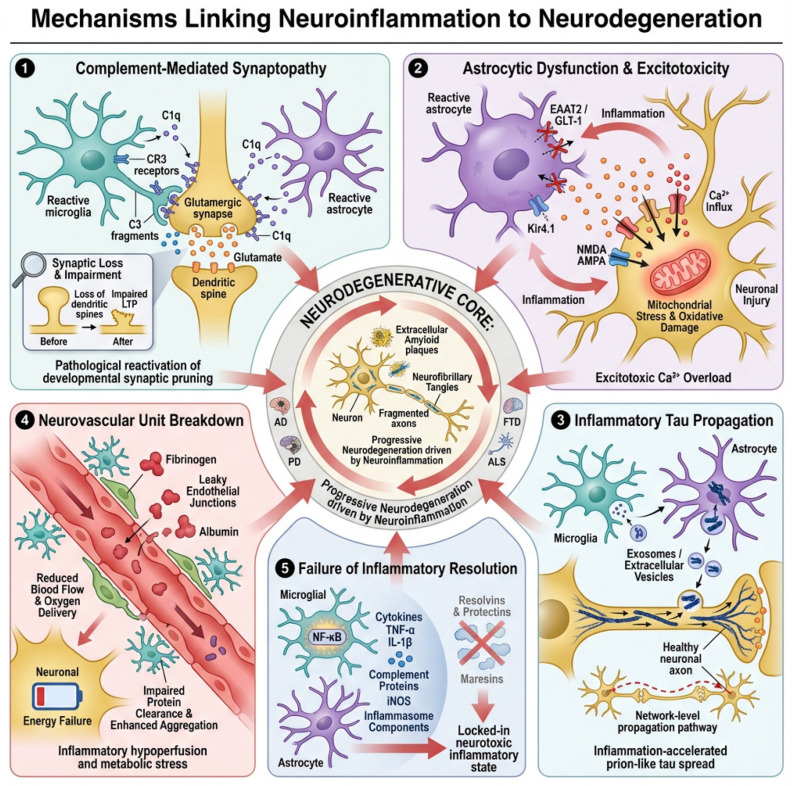
Mechanisms linking neuroinflammation to neurodegeneration. This schematic illustrates five interconnected, self-amplifying pathways through which chronic neuroinflammation drives structural and functional brain degeneration: (1) Complement-mediated synaptopathy: reactive microglia and astrocytes upregulate C1q, initiating the classical complement cascade and C3 opsonization of synapses, which are subsequently eliminated by CR3-expressing microglia, leading to early loss of connectivity, dendritic spine retraction, and impaired long-term potentiation. (2) Astroglial dysfunction and excitotoxicity: inflammatory cytokines downregulate astrocytic glutamate transport (EAAT2/GLT-1) and disrupt K^+^ buffering via Kir4.1, resulting in glutamate accumulation, excessive Ca^2+^ influx, oxidative stress, and neuronal hyperexcitability. (3) Glia-facilitated tau propagation: activated microglia and astrocytes internalize and release misfolded tau in extracellular vesicles, while inflammatory remodeling of synaptic and axonal membranes increases tau entry and spread across neural circuits. (4) Neurovascular unit failure: endothelial dysfunction, blood–brain barrier breakdown, pericyte loss, and impaired capillary regulation cause chronic hypoperfusion, metabolic stress, and enhanced amyloid and tau pathology. (5) Failed resolution of inflammation: diminished pro-resolving lipid signaling permits persistent NF-κB activation, sustaining toxic glial phenotypes and perpetuating synaptic, neuronal, and vascular damage. Together, these convergent mechanisms convert neuroinflammation into a progressive, network-level neurodegenerative process.

Without effective resolution, neuroinflammation becomes a self-maintaining state that continuously damages synapses, neurons, and blood vessels. Neurodegeneration thus emerges not simply from injury but from the brain’s inability to shut down its own immune response, as shown in Figure 3.

## 4. Neuroinflammatory Biomarkers in Alzheimer’s Disease: A Translational Perspective

As neuroinflammation has emerged as a central driver of Alzheimer’s disease (AD) pathogenesis, there has been an increasing effort to develop biomarkers that capture its presence, intensity, and biological meaning in living patients. Unlike amyloid-β and tau, which primarily reflect protein aggregation, inflammatory biomarkers provide a dynamic window into disease activity, progression, and therapeutic response. These markers span multiple biological compartments, including cerebrospinal fluid (CSF), plasma, and neuroimaging, and together allow the construction of a multidimensional picture of glial and vascular pathology [82].

### 4.1. Fluid Biomarkers of Glial Activation and Immune Signaling

One of the most robust biomarkers of astrocytic reactivity in AD is glial fibrillary acidic protein (GFAP). CSF and plasma GFAP levels rise early in the disease, even during preclinical stages, and correlate strongly with amyloid burden and white-matter injury. GFAP reflects astrocyte hypertrophy and cytoskeletal remodeling, serving as a proxy for astrocytic engagement with pathological stress [83].

Soluble TREM2 (sTREM2) has emerged as a key marker of microglial activation. TREM2 is a receptor involved in phagocytosis, lipid sensing, and survival signaling in microglia. Its soluble ectodomain, released into CSF and plasma, increases during the transition from amyloid deposition to tau-mediated neurodegeneration. Importantly, sTREM2 does not simply reflect inflammation but also captures microglial attempts to respond to and contain pathology [84,85].

YKL-40 (also known as CHI3L1) is a glycoprotein produced mainly by reactive astrocytes and, to a lesser extent, by microglia. Elevated YKL-40 levels correlate with tau pathology, cortical thinning, and cognitive decline, making it a useful marker of chronic glial activation and tissue remodeling [86,87].

Pro-inflammatory cytokines such as interleukin-6 (IL-6) and tumor necrosis factor-α (TNF-α) reflect active immune signaling within the central nervous system. Although their absolute concentrations are low and variable, sustained elevation in CSF or plasma indicates a pro-inflammatory milieu that promotes synaptic dysfunction, blood–brain barrier permeability, and neuronal stress [88,89].

Complement components (C1q, C3, C4) and chemokines such as CCL2 (MCP-1) and CXCL10 provide additional resolution. They report on immune recruitment, synapse tagging, and inflammatory amplification, linking molecular pathology to network-level synaptic loss [90,91].

Together, these fluid biomarkers allow clinicians and researchers to distinguish between amyloid-driven pathology and immune-mediated neurodegeneration, opening the door to patient stratification and personalized therapeutic strategies.

### 4.2. Neuroimaging of Glial and Vascular Pathology

Neuroimaging provides a spatially resolved and longitudinal view of neuroinflammation. The most established technique is TSPO-PET imaging, which detects the 18 kDa translocator protein expressed by activated microglia and reactive astrocytes. Increased TSPO binding is observed in regions affected by amyloid and tau pathology and correlates with cognitive decline and neurodegeneration [92,93].

However, TSPO imaging captures glial activation rather than function. It cannot distinguish whether microglia are protective, phagocytic, inflammatory, or degenerative. Nevertheless, it remains a powerful tool to visualize the geographic and temporal dynamics of immune activation in the living brain [94].

Magnetic resonance imaging (MRI) provides complementary information about the vascular and structural consequences of inflammation. Advanced MRI techniques can detect microhemorrhages, blood–brain barrier leakage, white-matter hyperintensities, and cortical microinfarcts, all of which reflect neurovascular unit dysfunction driven by inflammatory and oxidative stress. These vascular signatures are increasingly recognized as critical contributors to cognitive decline in Alzheimer’s disease [95,96].

### 4.3. Activation Versus Phenotype: A Critical Distinction

A central challenge in translating neuroinflammatory biomarkers into clinical practice is distinguishing glial activation from glial phenotype. Activation simply indicates that glial cells have responded to a stimulus, but it does not reveal whether their response is protective, reparative, or toxic.

For example, elevated sTREM2 may reflect beneficial microglial engagement with amyloid plaques, whereas high complement activity and TNF-α signaling indicate synapse-damaging inflammatory states. Similarly, astrocytic GFAP elevation may represent early protective remodeling or late-stage scar-like reactivity that blocks synaptic recovery [97,98].

Therefore, no single biomarker should be interpreted in isolation. Meaningful clinical insight emerges only from biomarker patterns that integrate astrocytic, microglial, cytokine, complement, and vascular signals. This multidimensional approach is essential for identifying patients in whom neuroinflammation is driving neurodegeneration and who may benefit from targeted immunomodulatory or pro-resolving therapies.

Despite significant progress in the development of neuroinflammatory biomarkers, several practical and conceptual limitations must be explicitly acknowledged. A major challenge is substantial inter-individual variability in biomarker levels, which is influenced by genetic background (e.g., APOE and TREM2 variants), age, sex, vascular and metabolic comorbidities, and peripheral inflammatory status. This heterogeneity complicates the establishment of universal diagnostic thresholds, reduces cross-cohort reproducibility, and limits the straightforward longitudinal interpretation of biomarker changes within individual patients. Consequently, robust standardization protocols and biomarker-based stratification strategies will be essential to maximize clinical interpretability and translational utility [99,100,101].

Specificity also remains a critical limitation, particularly for TSPO-PET imaging. Although TSPO-PET provides valuable spatial information about glial activation, it does not differentiate between protective, reparative, or neurotoxic inflammatory phenotypes, nor is TSPO expression unique to Alzheimer’s disease. Elevated TSPO binding is observed in a wide range of neurodegenerative and neuroinflammatory conditions, which reduces disease specificity and complicates clinical interpretation. For this reason, TSPO-PET is best viewed as a complementary rather than standalone biomarker and should be integrated with fluid and molecular measures of neuroinflammation [102,103].

Finally, cost and accessibility pose significant barriers to clinical implementation. TSPO-PET and other advanced neuroimaging modalities are expensive, technically demanding, and largely confined to specialized research centers, limiting their feasibility for routine clinical use. Similarly, many fluid biomarkers rely on high-cost analytical platforms and specialized laboratory infrastructure that are not yet widely available in standard clinical settings, particularly in low- and middle-income regions. These logistical constraints highlight the need for more scalable, standardized, and cost-effective biomarker approaches before neuroinflammatory measures can be fully integrated into precision diagnostic and therapeutic frameworks for Alzheimer’s disease [104].

### 4.4. Translational Implications

**Figure 4:**
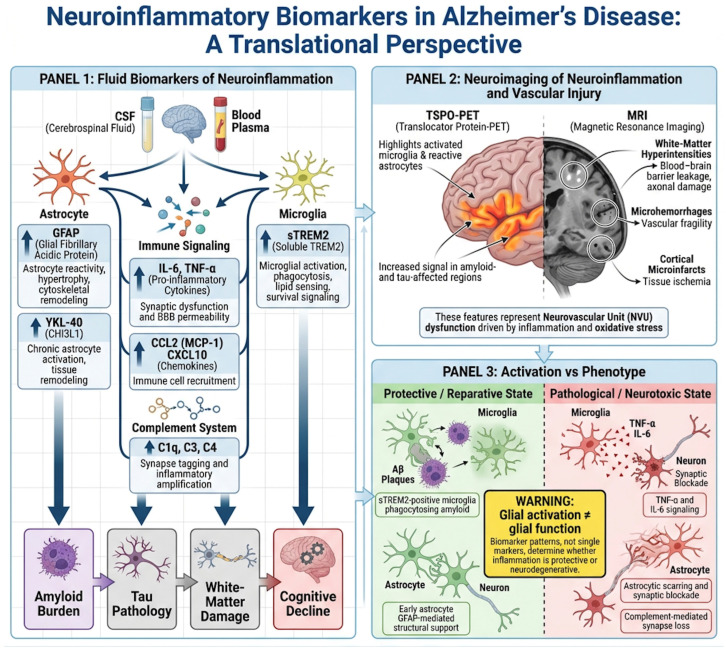
Neuroinflammatory biomarkers in Alzheimer’s disease across fluid and imaging modalities. This schematic summarizes key translational biomarkers of neuroinflammation in Alzheimer’s disease (AD), integrating fluid-based measures and neuroimaging approaches. In biofluids (CSF and plasma), markers of astrocytic reactivity (GFAP, YKL-40), microglial activation and response (sTREM2), pro-inflammatory cytokines (IL-6, TNF-α), and complement/chemokine signaling (C1q, C3, C4, CCL2, CXCL10) capture distinct but complementary aspects of glial activation, immune signaling, and synaptic vulnerability. These biomarkers collectively differentiate amyloid-associated changes from immune-mediated neurodegeneration and enable patient stratification. In parallel, neuroimaging modalities provide spatial and longitudinal information: TSPO-PET visualizes regional glial activation, while advanced MRI detects vascular and structural consequences of inflammation, including blood–brain barrier dysfunction, white-matter injury, and microvascular damage. Together, fluid and imaging biomarkers offer a multidimensional framework to characterize neuroinflammatory activity, track disease progression, and inform precision immunomodulatory therapies in AD.

Neuroinflammatory biomarkers are redefining Alzheimer’s disease as an immune-modulated disorder. By tracking glial biology, vascular integrity, and inflammatory tone, these markers bridge molecular pathology and clinical symptoms. They will be indispensable for selecting patients, monitoring therapeutic response, and ultimately for shifting AD treatment from amyloid removal to restoration of immune and homeostatic balance in the brain [105], as shown in Figure 4.

## 5. Therapeutic Implications: Why Anti-Inflammatory Strategies Have Failed and Determining Which Precision Approaches Hold Promise

Despite overwhelming evidence that neuroinflammation drives Alzheimer’s disease (AD) progression, clinical trials of anti-inflammatory therapies have largely failed. This apparent paradox does not invalidate the inflammatory hypothesis; rather, it reflects a mismatch between the complexity of brain immune biology and the simplistic nature of the interventions that have been tested. Alzheimer’s disease is not driven by a single inflammatory pathway but by a shifting landscape of glial phenotypes, vascular dysfunction, and failed resolution. Effective therapies must therefore be timed, targeted, and biologically informed [4].

An additional challenge for therapeutic intervention lies in the intrinsic complexity and context-dependence of immune signaling pathways in the brain. Neuroinflammatory responses are not uniformly deleterious, and several pathways exhibit stage- and phenotype-specific effects. Interferon (IFN) signaling provides a clear example of this duality [106]. While IFN pathways can contribute to host defense and early immune coordination, sustained or maladaptive IFN activation has been shown in experimental models to impair microglial phagocytic capacity, promote synaptic dysfunction, and exacerbate neurodegeneration [107]. Conversely, selective attenuation of IFN signaling can restore more homeostatic microglial responses and improve pathological outcomes in mouse models [108]. These observations highlight that effective therapeutic strategies must move beyond broad immunosuppression toward precision modulation of specific inflammatory pathways, tailored to disease stage and immune phenotype.

### 5.1. Why General Anti-Inflammatory Drugs Have Failed

Non-steroidal anti-inflammatory drugs (NSAIDs) were among the first agents tested based on epidemiological evidence suggesting reduced AD risk in chronic users. However, randomized trials have produced inconsistent or negative results.

The reasons are now clear. NSAIDs primarily inhibit cyclooxygenase pathways and prostaglandin synthesis, which are only a small part of the inflammatory network in the brain. More importantly, neuroinflammation in AD is not uniformly harmful. Early microglial and astrocytic responses can be protective by clearing amyloid, supporting synapses, and maintaining metabolic homeostasis. Broad suppression of inflammation can therefore impair beneficial immune functions while failing to block the toxic ones [109,110,111,112].

Timing is also critical. NSAIDs may only be beneficial before neurodegeneration is established, when inflammation is still compensatory. Once glial cells enter a chronic, self-amplifying state, these drugs are largely ineffective. Finally, patient heterogeneity means that only a subset of individuals may have inflammation-driven disease, further diluting trial outcomes.

### 5.2. Precision Immunomodulation as the New Paradigm

The future of neuroinflammatory therapy lies not in suppression but in reprogramming specific pathways that drive pathology.

#### 5.2.1. TREM2 and APOE Modulation

An additional challenge for therapeutic intervention lies in the intrinsic complexity and heterogeneity of immune signaling pathways in the brain. Not all inflammatory cascades contribute equally to neurodegeneration, and some may exert stage- and context-dependent effects. Interferon (IFN) signaling provides a clear example of this duality. While IFN pathways can participate in host defense and early immune coordination, sustained or maladaptive IFN activation has been shown in experimental models to impair microglial phagocytic capacity, promote synaptic dysfunction, and exacerbate neurodegeneration [113]. Conversely, selective attenuation of IFN signaling can restore more homeostatic microglial responses and improve pathological outcomes in mouse models [114]. These findings reinforce the need for precision immunomodulation strategies that target specific inflammatory pathways at defined disease stages, rather than broadly suppressing immune activity.

Microglial responses to amyloid and tau are strongly shaped by TREM2 and APOE signaling. Loss-of-function TREM2 variants impair plaque containment and accelerate neurodegeneration, while APOE4 biases microglia toward a pro-inflammatory, lipid-dysregulated phenotype. Therapeutic strategies that enhance protective TREM2 signaling or normalize APOE-dependent lipid handling aim to shift microglia from a neurotoxic to a reparative state [115,116].

#### 5.2.2. NLRP3 Inflammasome and IL-1β Inhibition

The NLRP3 inflammasome is a major driver of chronic neuroinflammation in AD. Activated by amyloid, tau, mitochondrial damage, and oxidative stress, NLRP3 promotes IL-1β release, synaptic dysfunction, and microglial toxicity. Targeted inhibition of NLRP3 or IL-1β offers a way to dampen the most destructive inflammatory outputs without disabling beneficial immune surveillance [117,118].

#### 5.2.3. Complement Inhibition to Prevent Synaptopathy

Blocking C1q or C3 prevents pathological synapse elimination by microglia while preserving the ability to clear debris and aggregates. This approach directly targets the mechanism that links inflammation to cognitive decline—loss of synaptic connectivity—rather than neuronal death [119,120].

Several immunomodulatory strategies have advanced into clinical or preclinical evaluation. TREM2-activating antibodies aim to enhance protective microglial responses, while complement inhibitors targeting C1q or C3 are being explored to prevent pathological synapse loss [121]. Inhibition of the NLRP3–IL-1β axis has shown promising results in experimental models, and senescence-targeted approaches are under active investigation [122].

However, immune modulation in the central nervous system carries inherent risks. Excessive suppression of microglial activity may impair amyloid clearance, increase susceptibility to infection, or disrupt essential homeostatic functions [13]. Conversely, overstimulation of immune pathways could exacerbate neuronal stress and synaptic damage. The dual role of glial cells—protective in early stages and potentially deleterious in chronic disease—highlights the importance of timing, disease stage, biomarker-guided stratification, and genetic background in therapeutic design [123].

Future trials will require integrated inflammatory biomarkers and precision patient selection to balance efficacy and safety.

### 5.3. Targeting the Neurovascular Unit

Restoring blood–brain barrier integrity and microvascular function is emerging as a powerful anti-neurodegenerative strategy. Therapies aimed at protecting endothelial cells, stabilizing pericytes, and restoring astrocytic endfeet can reduce immune cell infiltration, improve cerebral perfusion, and limit inflammatory amplification. In many patients, vascular inflammation may be the upstream driver of both amyloid accumulation and glial activation [124].

This therapeutic concept encompasses multiple mechanistic strategies aimed at stabilizing the structural and functional integrity of the neurovascular interface. Pharmacological approaches targeting endothelial inflammation—such as inhibitors of adhesion molecules, cytokine signaling pathways, and oxidative stress mediators—have demonstrated the capacity to reduce leukocyte infiltration and preserve tight junction integrity in experimental models [125]. Agents that enhance nitric oxide bioavailability or modulate vascular tone may additionally restore cerebral perfusion and reduce hypoxia-driven inflammatory signaling, thereby interrupting feed-forward cycles linking vascular dysfunction to glial activation [126].

Pericyte preservation represents another promising avenue. Loss of pericyte function contributes to blood–brain barrier leakage, capillary dysregulation, and impaired clearance of neurotoxic proteins. Experimental therapies aimed at stabilizing platelet-derived growth factor receptor-β signaling, modulating angiogenic pathways, or preventing pericyte apoptosis have shown neuroprotective effects in preclinical systems [127,128,129]. Together, these approaches suggest that targeting the neurovascular unit is not merely supportive therapy but may act upstream of both amyloid accumulation and neuroinflammatory amplification, positioning vascular stabilization as a disease-modifying strategy rather than a symptomatic intervention [130].

### 5.4. Pro-Resolving Therapies: Restoring Immune Balance

One of the most promising new directions is the use of specialized pro-resolving mediators (SPMs), including resolvins, protectins, and maresins. These lipid mediators do not suppress immunity; instead, they actively terminate inflammation, promote debris clearance, and stimulate tissue repair [81].

In Alzheimer’s disease, SPM pathways are deficient, allowing NF-κB-driven inflammation to persist indefinitely. Restoring pro-resolving signaling offers a fundamentally different therapeutic logic: not blocking inflammation but completing it [131].

### 5.5. Senescence-Targeted Therapies

If cellular senescence is included in the disease context, it offers another layer of precision therapy. Senescent microglia, astrocytes, and endothelial cells adopt a pro-inflammatory secretory phenotype that sustains neurodegeneration. Senolytic drugs remove these dysfunctional cells, while senomorphic agents suppress their toxic signaling without killing them. Both approaches aim to reset the inflammatory environment of the aging brain [132].

Senolytic agents act by selectively inducing apoptosis in senescent cells through the disruption of pro-survival signaling networks that are preferentially upregulated in these cells. Many senescent glial and endothelial populations rely on anti-apoptotic pathways involving BCL-2 family proteins, PI3K/AKT signaling, or p53 regulatory circuits [133]. Pharmacological inhibitors targeting these pathways can trigger cell death specifically in senescent populations while sparing healthy cells, thereby reducing the burden of the senescence-associated secretory phenotype (SASP), which is a major driver of chronic neuroinflammation and tissue dysfunction [134,135].

Experimental studies have demonstrated that the elimination of senescent glial cells can attenuate inflammatory cytokine production, improve synaptic integrity, and enhance cognitive performance in animal models of neurodegeneration [136]. In parallel, senomorphic compounds—which suppress SASP signaling without eliminating cells—offer an alternative strategy for modulating senescence-associated pathology with potentially lower toxicity [137]. Together, these complementary approaches support a model in which targeting cellular senescence restores tissue homeostasis by removing or reprogramming dysfunctional cellular populations that perpetuate inflammatory signaling within the aging brain.

#### Integrated Therapeutic Vision

**Figure 5:**
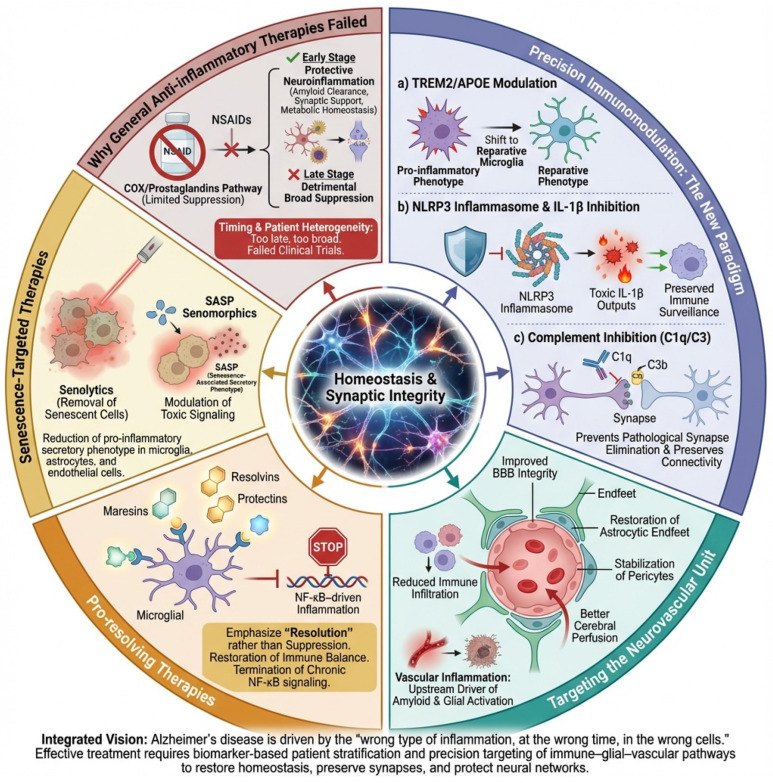
Therapeutic strategies targeting neuroinflammation in Alzheimer’s disease. This diagram highlights the differences between unsuccessful broad-spectrum anti-inflammatory methods and advanced precision immunomodulatory strategies in the context of Alzheimer’s disease (AD). Left, general anti-inflammatory therapies (e.g., NSAIDs) are shown to inadequately address the complex, dynamic, and cell-specific nature of neuroinflammation, often suppressing beneficial glial functions and being ineffective once chronic inflammation is established. Center, precision immunomodulation strategies aim to reprogram rather than suppress immunity, including the following: (a) modulation of TREM2 and APOE signaling to shift microglia toward a reparative phenotype; (b) targeted inhibition of the NLRP3 inflammasome/IL-1β axis to reduce toxic inflammatory outputs; and (c) complement (C1q/C3) inhibition to prevent pathological synapse loss while preserving debris clearance. Right, complementary approaches target the neurovascular unit to restore blood–brain barrier integrity and reduce vascular-driven inflammation, promote resolution of inflammation via specialized pro-resolving mediators (SPMs), and eliminate or modulate senescent glial and vascular cells through senolytic or senomorphic therapies. Collectively, the figure illustrates a shift from non-specific immunosuppression to biomarker-guided, pathway-specific interventions designed to restore immune balance, preserve synapses, and protect neuronal networks in AD.

The failure of past anti-inflammatory trials reflects a failure of conceptual framing. Alzheimer’s disease is not caused by “too much inflammation,” but by the wrong kind of inflammation at the wrong time in the wrong cells. The future lies in stratifying patients using neuroinflammatory biomarkers and targeting specific immune-glial-vascular pathways with precision therapies designed to restore homeostasis, preserve synapses, and protect the brain’s fragile ecosystems, Figure 5.

## 6. Discussion

The present context positions Alzheimer’s disease (AD) not as a disorder driven by isolated protein aggregates but as a failure of neuroimmune homeostasis in which amyloid-β (Aβ), tau, vascular dysfunction, and aging converge through chronic inflammation. This perspective resolves several long-standing paradoxes in AD research: the weak correlation between plaque load and cognitive decline, the heterogeneous clinical trajectories, and the disappointing outcomes of therapies aimed solely at amyloid or tau removal.

Within this framework, neuroinflammation emerges as a systems-level integrator of molecular pathology. Aβ oligomers behave as damage-associated molecular patterns (DAMPs) that engage microglial and astrocytic pattern-recognition receptors, triggering inflammasome activation, cytokine release, and oxidative stress [138,139]. These immune responses, initially protective, become maladaptive with persistent stimulation and age-related immune priming, converting localized protein misfolding into widespread network dysfunction. This model explains why amyloid deposition alone does not predict neurodegeneration: it is the interpretation of amyloid by the innate immune system, not its absolute quantity, that determines disease trajectory. Genetic risk factors such as APOE4 and TREM2 variants lower the threshold for immune activation and impair resolution, thereby magnifying the inflammatory response to otherwise modest Aβ burdens [140].

Although this review emphasizes neuroimmune dysregulation as a central integrator of Alzheimer’s disease (AD) pathology, several complementary hypotheses warrant consideration. Mitochondrial and metabolic models propose that impaired bioenergetics, oxidative stress, and insulin resistance may precede and contribute to amyloid and tau pathology [141]. Vascular hypotheses similarly suggest that chronic hypoperfusion, endothelial dysfunction, and blood–brain barrier breakdown represent early drivers of neuronal vulnerability [142].

Furthermore, the existence of cognitively normal individuals with substantial amyloid burden challenges a strictly amyloid-centric model and highlights the importance of resilience factors such as synaptic reserve, immune regulation, and vascular integrity [143]. In parallel, the limited cognitive impact observed in some anti-amyloid clinical trials suggests that amyloid removal alone may not be sufficient once inflammatory and synaptic cascades are established.

Rather than contradicting the neuroimmune framework, these models may converge mechanistically. Metabolic stress, mitochondrial dysfunction, vascular impairment, and protein aggregation all activate innate immune pathways. Neuroinflammation may therefore function as the biological interface through which diverse upstream insults are translated into synaptic dysfunction and network collapse [144].

At the synaptic level, complement-mediated synaptopathy reframes AD as a disease of connectivity collapse rather than primary neuronal loss. Reactivation of developmental pruning pathways (C1q–C3–CR3) leads to the selective elimination of glutamatergic synapses before neurons die, tightly correlating with cognitive impairment. This explains why clinical symptoms emerge long before extensive neuronal loss is detectable and why therapies that preserve synapses may be more impactful than those aimed at late-stage neuroprotection [63,145].

Glial dysfunction further links inflammation to neuronal injury. Reactive astrocytes lose their ability to buffer glutamate and potassium, creating a state of chronic network hyperexcitability and mitochondrial overload. This ionic and metabolic collapse provides a mechanistic bridge between immune activation and excitotoxicity, and the consistent downregulation of EAAT2 across AD and ALS underscores the universality of this glial-mediated vulnerability [11,146].

Neuroinflammation also acts as a catalyst of tau propagation. Rather than containing tau pathology, activated microglia and astrocytes facilitate its spread via extracellular vesicles and cytokine-driven membrane destabilization, positioning inflammation as a driver of prion-like network degeneration and explaining the stereotyped anatomical progression of tau pathology and its acceleration in inflammatory states [147,148].

At the vascular level, damage to the neurovascular unit introduces a metabolic dimension to the neuroimmune model. Blood–brain barrier breakdown, capillary stalls, and hypoperfusion deprive neurons of oxygen and glucose while amplifying immune cell infiltration and oxidative stress. This triad—proteinopathy, inflammation, and vascular failure—creates a self-reinforcing loop that locks the brain into progressive degeneration [149,150].

From a translational perspective, neuroinflammatory biomarkers such as GFAP, sTREM2, YKL-40, cytokines, complement factors, and TSPO-PET imaging provide a dynamic window into disease activity rather than static pathology. Their greatest value lies in multidimensional patterns that distinguish protective from toxic glial states, enabling patient stratification and precision therapy [151].

The failure of broad anti-inflammatory therapies such as NSAIDs reflects the functional heterogeneity of inflammation. Early glial responses are beneficial, whereas later responses become destructive; blunt suppression disrupts protective immunity while leaving toxic pathways intact. This highlights the need for therapies that reprogram rather than silence the brain’s immune system [152].

Accordingly, precision immunotherapy targeting TREM2/APOE signaling, NLRP3–IL-1β, complement, the neurovascular unit, senescence, and especially pro-resolving lipid mediators (SPMs) represents a paradigm shift from blocking inflammation to completing and resolving it. This strategy aligns with the biology of tissue repair and offers a rational path to preserving synapses and network integrity [153,154].

## 7. Conclusions

Alzheimer’s disease emerges from this work as a disorder of failed neuroimmune regulation, not merely of amyloid and tau accumulation. Chronic, unresolved inflammation transforms protein aggregates into a cascade of synaptic loss, network instability, tau propagation, and vascular-metabolic failure. Cognitive decline reflects the collapse of neural connectivity driven by complement-mediated pruning, astrocytic dysfunction, and microglial toxicity—processes orchestrated by a dysregulated immune system.

Neuroinflammatory biomarkers now allow us to visualize this immune landscape in living patients, enabling stratification and the rational deployment of precision immunomodulatory and pro-resolving therapies. The future of AD treatment lies not in removing proteins in isolation but in restoring immune balance, protecting synapses, and stabilizing the brain’s cellular ecosystems.

## Data Availability

No new data were created.

## Abbreviations

The following abbreviations are used in this manuscript:

Aβ	Amyloid-β
AD	Alzheimer’s disease
APOE/APOE4	Apolipoprotein E/Apolipoprotein E ε4 allele
BBB	Blood–brain barrier
C1q, C3	Complement components 1q and 3
CR3	Complement receptor 3
CSF	Cerebrospinal fluid
DAMPs	Damage-associated molecular patterns
EAAT2 (GLT-1)	Excitatory amino acid transporter 2
GFAP	Glial fibrillary acidic protein
IL-1β	Interleukin-1 beta
IL-6	Interleukin-6
MRI	Magnetic resonance imaging
NF-κB	Nuclear factor kappa-light-chain-enhancer of activated B cells
NLRP3	NOD-, LRR- and pyrin domain-containing protein 3
NSAIDs	Non-steroidal anti-inflammatory drugs
PET	Positron emission tomography
RAGE	Receptor for advanced glycation end products
ROS/RNS	Reactive oxygen/nitrogen species
sTREM2	Soluble triggering receptor expressed on myeloid cells 2
SPMs	Specialized pro-resolving mediators
TLR	Toll-like receptor
TNF-α	Tumor necrosis factor alpha
TREM2	Triggering receptor expressed on myeloid cells 2
TSPO	18 kDa translocator protein
